# COVID-19 AND DEMOGRAPHIC PREDICTORS OF CAREGIVER BURDEN AND DEPRESSION

**DOI:** 10.1093/geroni/igad104.2145

**Published:** 2023-12-21

**Authors:** Julian Montoro-Rodriguez, Jennifer Ramsey, Dolores Gallagther-Thompson

**Affiliations:** University of North Carolina Charlotte, Charlotte, North Carolina, United States; Case Western Reserve University, Cleveland, Ohio, United States; Stanford, Stanford, California, United States

## Abstract

Family caregivers are at increased risk for negative impacts on their health including higher depression compared to non-caregivers. The COVID19 pandemic presented challenges to adults with health conditions and exacerbated the demands that caregivers faced to continue providing care, support, services, and managing negative emotions. Our team in North Carolina adapted an evidence-based psychoeducational program “Coping with Caregiving” developed from the REACH II studies, and created the “Caregiver Thrive, Learn & Connect” to assist caregivers to manage stress, learn skills and connect with others. Caregivers attended workshops early in 2021 up to 2023 (https://caregivertlc.org/). COVID-19 research shows that burden, social support, education, income and age are predictors of depression. Using the initial data (pre-program intervention, Time 1) from the Caregiver TLC intervention we examined the impact of COVID-19 on the caregivers’ level of burden and depression. Our sample includes 107 informal caregivers (age mean=63; female=84%; White=66%; Black 33%; living with spouse=68%; and median income of $75-$89K). Predictors of depression were regressed on the caregiver burden, the COVID19 severity exposure (2021 vs. 2022/23), the caregiver age, race (Blacks), education, income and perceived health. We also explored predictors of burden. Results indicated moderate adjusted R2 for depression (.42%) and burden (.16%). COVID-19 was a significant predictor for depression (-.33), along with the caregivers’ health (-.20). Burden was negatively impacted by the caregivers’ health (-.22), age (-.20) and for Blacks (-.20). Caregivers’ depression was impacted by the early stage of COVID19 in addition to their level of burden.

